# Arginine Catabolic Mobile Elements in Livestock-Associated Methicillin-Resistant Staphylococcal Isolates From Bovine Mastitic Milk in China

**DOI:** 10.3389/fmicb.2018.01031

**Published:** 2018-05-16

**Authors:** Chao Tong, Zhaowei Wu, Xin Zhao, Huping Xue

**Affiliations:** ^1^College of Animal Science and Technology, Northwest A&F University, Yangling, China; ^2^Department of Animal Science, McGill University, Montreal, QC, Canada

**Keywords:** livestock-associated staphylococci, arginine catabolic mobile element, SCC*mec*, heavy metal tolerance, methicillin resistance

## Abstract

The arginine catabolic mobile element (ACME) facilitates colonization of staphylococci on skin and mucous membranes by improving their tolerances to polyamines and acidic conditions. ACME is inserted in tandem with the SCC*mec* element and *Staphylococcus epidermidis* has been proposed to be a reservoir of ACME for other staphylococci. In this study, we investigated the existence of ACME in 146 staphylococcal isolates from mastitic milk and found 21 of them carried ACME. Almost half of the investigated *S. epidermidis* isolates contained the element. The whole genome of a *S. epidermidis* strain Y24 with ACME was further sequenced and the ACME-SCC*mec* composite island was assembled. This composite island is 81.3 kb long and consisted of 77 ORFs including a methicillin resistance gene *mecA*, a type II’ ACME gene cluster, a virulence gene *pls* and eight heavy metal tolerance genes. Wide existence of ACME in livestock-associated staphylococci from this study and a potential risk of spreading ACME among different staphylococcal species warrant close monitoring and further studies.

## Introduction

The opportunistic pathogen *Staphylococcus epidermidis* is a permanent and common commensal bacterium of skin and mucosa. As the most important cause of infections on indwelling medical devices (Otto, 2009), *S. epidermidis* has become the third most common source of nosocomial infections (Sievert et al., 2013; Rupp, 2014). Besides its pathogenicity, *S*. *epidermidis* is proposed to be a main reservoir of antimicrobial resistance genes and virulence genes for *Staphylococcus aureus* (Otto, 2013), by horizontal transferring of mobile genetic elements such as Staphylococcal chromosomal cassette *mec* (SCC*mec*) and arginine catabolic mobile element (ACME) (Diep et al., 2006; Thurlow et al., 2013; Xue et al., 2017).

Arginine catabolic mobile element is inserted in tandem with the SCC*mec* (ACME-SCC*mec* composite island) and contains an *arc* cluster, an *opp*-3 cluster and *speG* gene. ACME allotypes are defined by the presence or absence of the *arc* and *opp*-3 gene clusters (type I, *arc*^+^ *opp*-3^+^; type II, *arc*^+^ *opp*-3^-^; type III, *arc*^-^ *opp*-3^+^). Carriage of ACME also improves the capacity and fitness of *S. aureus* to colonize the skin and mucosa (Diep et al., 2008). The *arc* cluster mediates the arginine-deiminase pathway that converts L-arginine to L-ornithine for production of both ATP and ammonia, the latter is responsible for enhanced acid tolerance of staphylococci on human skin (Alonzo and Torres, 2013; Thurlow et al., 2013). The *speG* encodes a spermine/spermidine N-acetyltransferase (SpeG), which detoxifies polyamines that are produced by all living organisms. SpeG allows the strains to evade the toxicity of polyamines secreted on the human skin and to potentiate its colonization and infection.

Arginine catabolic mobile element was first identified in *S. aureus* USA300 in 2006 (Diep et al., 2006) and was proposed to contribute to successful spread of this community clone in North America and Europe (Talan et al., 2011; Thurlow et al., 2012; Glaser et al., 2016). ACME has been reported in the staphylococcal isolates from humans in several Asian countries. The earliest report of the ACME element in Asia was in Japan, 2007 (Shibuya et al., 2008), and an increasing trend was observed afterward (Onishi et al., 2013; Urushibara et al., 2016; Aung et al., 2017). In addition to Japan, 38.7% of 360 *S. aureus* sequence type 239-like isolates in Australia (obtained between 1997 and 2008) was reported to carry an ACME (Espedido et al., 2012), and 12.7% ST239 strains isolated between 2000 and 2010 in Singapore also carried the ACME element (Hon et al., 2013). A later evolutionary study found that the local clone ST239 in Singapore began to increase from 2007 onward after they acquired ACME (Hsu et al., 2015). The first case of ACME element in Korea was reported in 2016 (Jung et al., 2016). The presence of ACME in the staphylococcal isolates from animals was much less reported. A previous study reported that almost half of *S. epidermidis* isolates (12/22) in pigs contained the *arc* cluster (Argudín et al., 2015). In addition, McManus et al. (2015) detected the ACME in staphylococcal isolates from companion animals. However, the report about ACME in staphylococcal isolates from both humans and animals in China is scarce.

In this study, we found that the ACME element widely existed in livestock isolated staphylococcal isolates, especially in *S. epidermidis*. The whole genome of an *S. epidermidis* isolate Y24 was then sequenced. An ACME-SCC*mec* composite island with a length of 81.3 kb was found in the genome of Y24, conferring this bacterium with β-lactam antibiotics resistance and heavy metal resistance. Based on comparative analyses, we found the ACME-SCC*mec* composite island in Y24 was phylogenetically close to the element of *S. aureus* strains in Germany and Denmark.

## Materials and Methods

### Bacterial Isolates and ACME Screening

In the year of 2015, a total of 146 staphylococcal isolates were collected from Holstein cow milk with clinical mastitis at three local dairy farms in Shaanxi province, Northwest China. They were screened for the presence of ACME-*arcA* and ACME-*opp3AB* genes by PCR (Diep et al., 2008). Primer sequences are listed in the **Supplementary Table S1**. All PCR products were sequenced by Sanger sequencing.

### Whole Genome Sequencing

*Staphylococcus epidermidis* strain Y24 was identified by 16S rRNA sequencing and whole genome sequencing (WGS). The genomic DNA library with an average insertion size of 500 bp was prepared and paired-end sequencing was performed on the Illumina Hiseq 2000 platform. In order to obtain high quality reads for assembly, low quality reads and adaptor sequences were filtered by readfq.v5 (The Beijing Genomics Institute, China). WGS sequencing reads were assembled by the SOAPdenovo software (version 2.04) using the available genome sequence of *S. epidermidis* ATCC 12228 as reference guided assembling (Li et al., 2010).

### Assembly and Identification of SCC Elements in Y24

To find SCC elements in Y24 strain, each scaffold of Y24 was searched for *orfX* region, *mecA* gene and *ccr* gene complex using the BLASTN (version 2.6.0+) software. All positive scaffolds were aligned against the SCC*mec* IVa of *S. aureus* strain R99 (GenBank accession no. KF234240.1) to verify their genetic organization. All the gaps were closed using PCR amplification. Gene prediction and functional annotation were accomplished using the Rapid Annotations Subsystems Technology (RAST) server (version 2.0) (Aziz et al., 2008). Then, the nucleotide sequences of the predicted genes were analyzed by BLAST against the NCBI nucleotide collection (nr/nt) database and the non-redundant protein sequence database. Primer sequences are listed in the **Supplementary Table S1**.

### PCRs for Detecting Excision of SCC Elements and ACME

PCR reactions were performed for investigating excision of SCC elements, ACME and their composite islands by detecting the extrachromosomal circular intermediates, according to the method described by Ito et al. (Ito et al., 1999). A positive PCR strand could be observed if an excision occurs, by using the primer pairs of 1F+1R, 2F+2R, 3F+3R, and 4F+4R for detecting single SCC elements, or the primer sets 2F+1R, 3F+1R, 4F+1R, 3F+2R, 4F+2R, and 4F+3R for detecting composite SCC elements. The primer sequences are listed in **Supplementary Table S1**. Y24 strain was grown in the brain heart infusion (BHI) broth. Extracted genomic DNA of Y24 from logarithmic phase cultures was used as template. The products were then cloned into a pEASY-Blunt Zero vector (Transgen, China) for Sanger sequencing.

### Minimum Inhibitory Concentrations (MIC) Determinations

Minimum inhibitory concentrations for the antimicrobial agents were performed by the standard agar dilution method according to Clinical and Laboratory Standards Institute (CLSI) guidelines in the year of 2015 (Clinical Laboratory Standards Institute [CLSI], 2015). Because oxacillin-susceptible *mecA*-positive Staphylococci can be converted to oxacillin-resistant phenotype under oxacillin induction (Liu et al., 2016), strain Y24 was subjected to oxacillin induction by serial passages under oxacillin gradients as 0, 0.5, 1, 2, and 4 mg/L from day 0 to day 4. MICs of heavy metal ions were determined as previously described (Xue et al., 2015). MHA (Mueller-Hinton agar) plates with different final concentrations of heavy metal ions were provided NaAsO_2_ for arsenic, ZnSO_4_⋅7H_2_O for zinc, CuSO_4_⋅5H_2_O for copper, Cd(NO_3_)_2_⋅4H_2_O for cadmium, Pb(CH_3_COO)_2_⋅3H_2_O for lead and HgCl_2_ for mercury.

### Bioinformatics Analyses

In order to determine phylogenetic relations among the ACME-SCC*mec* composite island in Y24 and other reported composite SCCs, the NCBI nucleotide blast software was used for genome alignments in the nr/nt and wgs databases with default parameters (accessed on May 12th, 2017). The query sequence was the identified 81.3 kb nucleotide sequence of ACME-SCC*mec* composite island in Y24. The comparison of different composite SCCs was conducted using BLASTN with the softwares Mauve (Darling et al., 2010) and Easyfig (Sullivan et al., 2011).

## Results

### Distribution of ACME Element in Livestock-Associated Staphylococci

In order to find potential distributions of ACME element in livestock-associated staphylococci, a total of 146 staphylococcal isolates from three dairy farms in Northwestern China were screened (**Table 1**). Approximately 14% (21/146) of them possessed ACME (9 for type I, 2 for type II or II’ and 10 for type III). Most of the ACME positive isolates (15/21) distributed in a same farm. All of the 9 type I ACME elements were located in *S. epidermidis*, while 10 type III ACME elements were distributed in four different staphylococcal species (**Table 1**). Eleven out of 24 *S. epidermidis* isolates contained ACME element, while no ACME was found in any *S. agnetis* or *S. warneri* isolates.

**Table 1:** Prevalence of ACME in strains isolated from Holstein cow milk samples.

Allotype	*S. epidermidis* (*n* = 24)	*S. haemolyticus* (*n* = 34)	*S. aureus* (*n* = 37)	*S. chromogenes* (*n* = 19)	*S. warneri* (*n* = 30)	*S. agnetis* (*n* = 2)	Total (*n* = 146)
I	9	0	0	0	0	0	9
II or II’	1	1	0	0	0	0	2
III	1	3	1	5	0	0	10

### The ACME-SCC*mec* Composite Island and Resistant Features of *S. epidermidis* Y24

**FIGURE 1:**
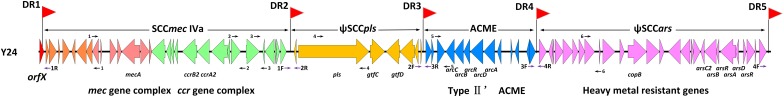
Genetic structure of the ACME-SCC*mec* composite island in *S. epidermidis* Y24. ORFs are shown as arrows indicating the transcription direction, and colors mean different gene fragments. Red flags (DR1 to DR5) indicate the locations of different integration site sequences. Thin purple arrows show the location of PCR primers for detecting the precise excision.

Because almost half of the investigated *S. epidermidis* isolates contained the ACME element, one of them, Y24, which was resistant to a range of antimicrobial drugs and heavy metals (**Tables 2**, **3**), was chosen for WGS. The WGS data of 399 Mb from Y24, giving approximately 140-fold genome coverage, were generated and assembled into 140 contigs and joined into 56 scaffolds. Among them, five scaffolds were identified as sections of the ACME-SCC*mec* composite island and six PCRs were performed to close the gaps between/in these scaffolds (**Figure 1** and **Supplementary Table S1**). Finally, the full length of ACME-SCC*mec* composite island of Y24 was determined to be 81.3 kb (GenBank accession no. KY849363). It consisted of an intact SCC*mec* element, an ACME element and two pseudo-SCC elements designated ψSCC*pls* and ψSCC*ars* (**Figure 1**). The SCC*mec* element (*orf2* to *orf30*) comprised a *mec* gene complex and a *ccrA2B2* gene complex and was classified as a type IVa SCC*mec* (International Working Group on the Classification of Staphylococcal Cassette Chromosome Elements, 2009; Wu et al., 2015). The ACME element (*orf38* to *orf51*) was classified as a type ACME II’, because it contained an *arc* gene cluster but lacked an *opp-3* cluster and several genetic components surrounding the *arc* gene cluster. Among 77 predicted open reading frames (ORFs) in the ACME-SCC*mec* composite island, 10 ORFs were resistance-associated genes (2 for antibiotics and 8 for heavy metals resistance), 6 ORFs were related to DNA transfer (4 insertion sequences and 2 recombinases) and 6 ORFs were related to the arginine-deiminase pathway (**Supplementary Table S2**). The composite island in Y24 included five integration site sequences including one in *orfX*.

**Table 2:** Results of susceptibility testing against antibiotics of Y24.

Strain (species)	NEO	CLI	OXA	VAN	KAN	CEF	TEC	HYG	CIP	STR	GEN	PEN	TMP
Y24 (*S. epidermidis*)	2	4	0.5	2	>256	64	2	>256	4	32	0.5	16	>256
RN4220 (*S. aureus*)	8	4	0.5	1	>256	16	1	>256	4	32	1	0.5	0.5

*NEO, neomycin; CLI, clindamycin; OXA, oxacillin; Van, vancomycin; KAN, kanamycin; CEF, cephalothin; TEC, teicoplanin; HYG, hygromycin; CIP, ciprofloxacin; STR, streptomycin; GEN, gentamicin; PEN, penicillin G, TMP, trimethoprim.*

**Table 3:** Results of susceptibility testing against heavy metal compounds of Y24.

Strains (species)	MIC (mM) of heavy metal					
	As	Cd	Pb	Cu	Zn	Hg
Y24 (*S. epidermidis*)	>8	0.125	>8	2	2	0.025
RN4220 (*S. aureus*)	0.015	0.015	>8	2	2	0.05

Y24 displayed multidrug resistance to kanamycin (MIC > 256 ug/ml), trimethoprim (MIC>256 ug/ml), hygromycin B (MIC>256 ug/ml), cephalothin (MIC = 64 ug/ml), streptomycin (MIC = 32 ug/ml) and penicillin G (MIC = 16 ug/ml). Though the MIC of Y24 to oxacillin was only 0.5 ug/ml, it displayed an oxacillin resistant phenotype (MIC = 64 ug/ml) after 4 days of low oxacillin inducing. The genome contains *blaZ* gene for resistance to penicillin and *msrA* and *mphC* genes for resistance to macrolide compounds. Genes *dfrA* and *drfG* in the genome contributed to trimethoprim resistance (**Supplementary Table S2**). The Y24 also displayed a high tolerance to arsenic (MIC>8 mM) and lead (MIC>8 mM). The *orf62* coding lead resistance and the *orf69* to *orf74* coding arsenic resistance could be responsible for the arsenic and lead tolerances of Y24 (**Supplementary Table S2**). The MICs for mercury, cadmium, zinc and copper ions were 0.025, 0.125, 2, and 2 mM, respectively, which may result from expression of *orf62* and *orf63* (**Table 2**).

### Comparative Analyses of the ACME-SCC*mec* Composite Island in Y24 With Those in Other Staphylococcal Strains

**FIGURE 2:**
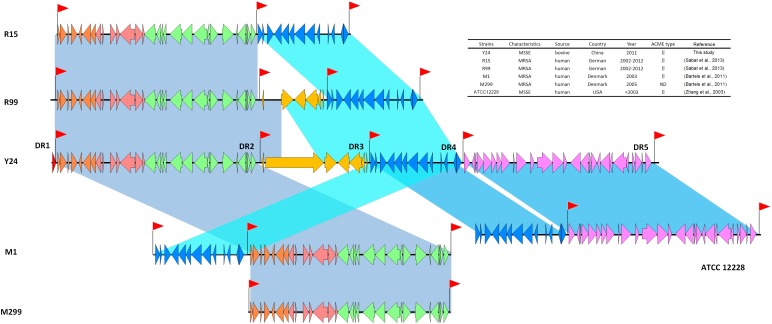
Comparative structure analyses of the ACME-SCC*mec* composite island in *S. epidermidis* Y24 against sequences of five different staphylococcal strains R15, R99, M1, M299, and ATCC 12228.

Thirty sequences from different staphylococcal strains with the highest similarities with the ACME-SCC*mec* composite island in Y24 were chosen for detailed analyses (**Supplementary Table S3**). Among them, the top five sequences showing the highest similarity scores (sequence identity>99%) with the ACME-SCC*mec* composite island in Y24 were those from strains M1, M299, R99, R15, and ATCC 12228. The accession numbers of them are HM030720, HM030721, KF234240, KF184643, and AE015929, respectively (**Figure 2**). Strain Y24 shared the SCC*mec* element (*orf2* to *orf30*) with R15, R99, M1, and M299. It also shared the ACME (*orf38* to *orf50*) with R15, R99, M1, and ATCC 12228. However, the heavy metal resistance region (*orf51* to *orf77*) was present only in Y24 and ATCC 12228, and absent in R15, R19, M1, or M299. All 30 strains investigated in this study contained a same defensive system as Type I restriction-modification system, which included three USA300 strains as USA300-ISMMS1, USA300_2014.C01, and UA-S391_USA300 (**Supplementary Table S3**).

Comparative analyses suggested that the SCC*mec* element or ACME in Y24 could be mobile. In order to check the possibility, PCRs were performed to detect extrachromosomal circular intermediates of SCC elements or ACME between direct repeats (DRs). Strong positive PCR amplicons were detected not only for the primer sets of 1F+1R, 2F+2R, 3F+3R, and 4F+4R, but also for the primer sets 2F+1R, 3F+1R, 4F+1R, 3F+2R, 4F+2R, and 4F+3R, respectively (**Supplementary Figure S1**). These results confirmed the formation of circular forms in Y24 for single SCC elements (SCC*mec*, ψSCC*pls*, ACME, or ψSCC*ars*) and the composite islands (SCC*mec*-ψSCC*pls*, SCC*mec*-ψSCC*pls*-ACME, etc.).

## Discussion

Sabat et al. (2015) reported ACME elements in two MRSA ST398 isolates from humans. Another study reported the *arc* cluster, one of the ACME components, existed in the *S. epidermidis* isolated from pigs (Argudín et al., 2015). Here, we report the presence of the ACME element in different staphylococcal species isolated from livestock, based on both the *arc* cluster and the *opp3* gene cluster. A previous study found that multiple virulence factors could be transferred between a bovine-originated staphylococci and a hospital-associated staphylococcal strain MRSA252 (Brody et al., 2008). Our recent studies also suggested possible occurrence of a horizontal gene transfer between staphylococci in bovine and staphylococci in humans (Xue et al., 2015, 2017). Based on the detection of extrachromosomal circular intermediates, we found the formation of circular forms in Y24 for both single and composite SCC elements. The large serine recombinases CcrA2B2 encoded in the SCC*mec* element of Y24 could be responsible for the formation of extrachromosomal circular intermediates. The circular form of ACME element in Y24 suggests the possibility of horizontal transfer of ACME from Y24 to other staphylococci. Thus, the high prevalence of ACME in livestock-associated staphylococcal isolates, especially in *S. epidermidis*, should be a major concern, since it could be transferred to human-associated staphylococci. In addition, an amplicon corresponding to the primers 1R+4F proved the ACME, SCC*mec* and several heavy metal resistant genes coexisted in an extrachromosomal circular, suggesting that they could be transferred as a whole to a new host. The CopB (encoded by *copB*, *orf62*) was related to copper and lead resistance. In addition, *arsB* gene (*orf70*) encodes an arsenic efflux pump protein and *arsA* (*orf72*) encodes arsenical pump-driving ATPase that could enhance the arsenic efflux capacity of the ArsB pump (Mukhopadhyay et al., 2002), giving Y24 strong resistance to arsenic (MIC>8 mM). The coexistence of antibiotic resistance genes as well as heavy metal resistance genes in the ACME-SCC*mec* composite island in Y24 could increase its survival both in contaminated environment and under antibiotic pressure (Xue et al., 2015).

The ACME-SCC*mec* composite island in Y24 contained many other genes such as a plasmin sensitive surface gene (*pls*, o*rf32*), a histidine kinase two-component system (*orf57* and *orf58*) and several heavy metal tolerance genes (*orf62*, *orf69* to *orf74*). Pls protein was a virulence factor in a mouse septic arthritis model (Josefsson et al., 2005). Recently, Bleiziffer et al. (2017) reported that Pls decreased the phagocytosis of *S. aureus*. The same study found that Pls was a glycoprotein and could promote biofilm formation of *S. aureus* (Bleiziffer et al., 2017). The glycosylation of Pls involved two glycosyltransferases GtfC and GtfD, which were also present in the composite island of Y24 (*orf33* and *orf34*). Co-existences of *pls*, *gtfC*, and *gftD* in Y24 could improve its virulence and resistance. Gene *orf58* encodes a two-component sensor histidine kinase, while gene *orf57* encodes a response regulator. Both genes were only found in three *S. epidermidis* strains (ATCC 12228, PM221 and SEI), depending on the NCBI nucleotide collection (nr/nt) database. What are the functions of the two component system in *S. epidermidis* strains needs to be further studied.

## Author Contributions

CT performed the experiments, analyzed the experiment data, and wrote the manuscript. ZW performed the experiments. HX and XZ designed the experiments, analyzed the result, and reviewed the manuscript.

## Conflict of Interest Statement

The authors declare that the research was conducted in the absence of any commercial or financial relationships that could be construed as a potential conflict of interest.

## Supplementary Material

FIGURE S1 PCR results confirming the formation of extrachromosomal circular intermediate using different primer sets.

TABLE S1 Oligonucleotides used in this work.

TABLE S2 Predicted ORFs in the SCC elements and ACME of *S. epidermidis* Y24.

TABLE S3 Defensive systems and origin of the strains.

## References

[B1] Alonzo F. III Torres V. J. (2013). A lesson in survival: *S. aureus* versus the skin. 13 3–5. 10.1016/j.chom.2013.01.001 23332150

[B2] Argudín M. A. Vanderhaeghen W. Butaye P. (2015). Antimicrobial resistance and population structure of *Staphylococcus epidermidis* recovered from pig farms in Belgium. 203 302–308. 10.1016/j.tvjl.2015.01.008 25676880

[B3] Aung M. S. Kawaguchiya M. Urushibara N. Sumi A. Ito M. Kudo K. (2017). Molecular characterization of methicillin-resistant *Staphylococcus aureus* from outpatients in Northern Japan: increasing tendency of ST5/ST764 MRSA-IIa with arginine catabolic mobile element. 23 616–625. 10.1089/mdr.2016.0176 27869532

[B4] Aziz R. K. Bartels D. Best A. A. DeJongh M. Disz T. Edwards R. A. (2008). The RAST Server: rapid annotations using subsystems technology. 9:75. 10.1186/1471-2164-9-75 18261238 PMC2265698

[B5] Bartels M. D. Hansen L. H. Boye K. Sorensen S. J. Westh H. (2011). An unexpected location of the arginine catabolic mobile element (ACME) in a USA300-related MRSA strain. 6:e16193. 10.1371/journal.pone.0016193 21283578 PMC3026799

[B6] Bleiziffer I. Eikmeier J. Pohlentz G. McAulay K. Xia G. Hussain M. (2017). The plasmin-sensitive protein pls in methicillin-resistant *Staphylococcus aureus* (MRSA) is a glycoprotein. 13:e1006110. 10.1371/journal.ppat.1006110 28081265 PMC5230774

[B7] Brody T. Yavatkar A. S. Lin Y. Ross J. Kuzin A. Kundu M. (2008). Horizontal gene transfers link a human MRSA pathogen to contagious bovine mastitis bacteria. 3:e3074. 10.1371/journal.pone.0003074 18728754 PMC2518619

[B8] Clinical Laboratory Standards Institute [CLSI] (2015). *Performance Standards for Antimicrobial Disk, and Dilution Susceptibility Tests for Bacteria Isolated from Animals; 3rd Informational Supplement*. Wayne, PA: CLSI.

[B9] Darling A. E. Mau B. Perna N. T. (2010). progressiveMauve: multiple genome alignment with gene gain, loss and rearrangement. 5:e11147. 10.1371/journal.pone.0011147 20593022 PMC2892488

[B10] Diep B. A. Gill S. R. Chang R. F. Phan T. H. Chen J. H. Davidson M. G. (2006). Complete genome sequence of USA300, an epidemic clone of community-acquired meticillin-resistant *Staphylococcus aureus*. 367 731–739. 10.1016/S0140-6736(06)68231-7 16517273

[B11] Diep B. A. Stone G. G. Basuino L. Graber C. J. Miller A. des Etages S. A. (2008). The arginine catabolic mobile element and staphylococcal chromosomal cassette mec linkage: convergence of virulence and resistance in the USA300 clone of methicillin-resistant *Staphylococcus aureus*. 197 1523–1530. 10.1086/587907 18700257

[B12] Espedido B. A. Steen J. A. Barbagiannakos T. Mercer J. Paterson D. L. Grimmond S. M. (2012). Carriage of an ACME II variant may have contributed to methicillin-resistant *Staphylococcus aureus* sequence type 239-like strain replacement in Liverpool Hospital, Sydney, Australia. 56 3380–3383. 10.1128/AAC.00013-12 22391530 PMC3370721

[B13] Glaser P. Martins-Simoes P. Villain A. Barbier M. Tristan A. Bouchier C. (2016). Demography and intercontinental spread of the USA300 community-acquired methicillin-resistant *Staphylococcus aureus* lineage. 7 e02183–15. 10.1128/mBio.02183-15 26884428 PMC4752609

[B14] Hon P. Y. Chan K. S. Holden M. T. Harris S. R. Tan T. Y. Zu Y. B. (2013). Arginine catabolic mobile element in methicillin-resistant *Staphylococcus aureus* (MRSA) clonal group ST239-MRSA-III isolates in Singapore: implications for PCR-based screening tests. 57 1563–1564. 10.1128/AAC.02518-12 23318798 PMC3591910

[B15] Hsu L. Y. Harris S. R. Chlebowicz M. A. Lindsay J. A. Koh T. H. Krishnan P. (2015). Evolutionary dynamics of methicillin-resistant *Staphylococcus aureus* within a healthcare system. 16:81. 10.1186/s13059-015-0643-z 25903077 PMC4407387

[B16] International Working Group on the Classification of Staphylococcal Cassette Chromosome Elements (2009). Classification of staphylococcal cassette chromosome mec (SCCmec): guidelines for reporting novel SCCmec elements. 53 4961–4967. 10.1128/AAC.00579-09 19721075 PMC2786320

[B17] Ito T. Katayama Y. Hiramatsu K. (1999). Cloning and nucleotide sequence determination of the entire mec DNA of pre-methicillin-resistant *Staphylococcus aureus* N315. 43 1449–1458. 10348769 10.1128/aac.43.6.1449 PMC89295

[B18] Josefsson E. Juuti K. Bokarewa M. Kuusela P. (2005). The surface protein Pls of methicillin-resistant *Staphylococcus aureus* is a virulence factor in septic arthritis. 73 2812–2817. 10.1128/IAI.73.5.2812-2817.2005 15845485 PMC1087342

[B19] Jung J. Song E. H. Park S. Y. Lee S. R. Park S. J. Sung H. (2016). Emergence of panton-valentine leucocidin-positive ST8-methicillin-resistant *Staphylococcus aureus* (USA300 clone) in Korea causing healthcare-associated and hospital-acquired bacteraemia. 35 1323–1329. 10.1007/s10096-016-2668-y 27209287

[B20] Li R. Zhu H. Ruan J. Qian W. Fang X. Shi Z. (2010). De novo assembly of human genomes with massively parallel short read sequencing. 20 265–272. 10.1101/gr.097261.109 20019144 PMC2813482

[B21] Liu P. Xue H. Wu Z. Ma J. Zhao X. (2016). Effect of bla regulators on the susceptible phenotype and phenotypic conversion for oxacillin-susceptible mecA-positive staphylococcal isolates. 71 2105–2112. 10.1093/jac/dkw123 27154864

[B22] McManus B. A. Coleman D. C. Deasy E. C. Brennan G. I. O’ Connell B. Monecke S. (2015). Comparative genotypes, staphylococcal cassette chromosome mec (SCCmec) genes and antimicrobial resistance amongst *Staphylococcus epidermidis* and *Staphylococcus haemolyticus* isolates from infections in humans and companion animals. 10:e0138079. 10.1371/journal.pone.0138079 26379051 PMC4574763

[B23] Mukhopadhyay R. Rosen B. P. Phung L. T. Silver S. (2002). Microbial arsenic: from geocycles to genes and enzymes. 26 311–325. 10.1111/j.1574-6976.2002.tb00617.x 12165430

[B24] Onishi M. Urushibara N. Kawaguchiya M. Ghosh S. Shinagawa M. Watanabe N. (2013). Prevalence and genetic diversity of arginine catabolic mobile element (ACME) in clinical isolates of coagulase-negative staphylococci: identification of ACME type I variants in *Staphylococcus epidermidis*. 20 381–388. 10.1016/j.meegid.2013.09.018 24113082

[B25] Otto M. (2009). *Staphylococcus epidermidis*–the ‘accidental’ pathogen. 7 555–567. 10.1038/nrmicro2182. 19609257 PMC2807625

[B26] Otto M. (2013). Coagulase-negative staphylococci as reservoirs of genes facilitating MRSA infection: Staphylococcal commensal species such as *Staphylococcus epidermidis* are being recognized as important sources of genes promoting MRSA colonization and virulence. 35 4–11. 10.1002/bies.201200112 23165978 PMC3755491

[B27] Rupp M. E. (2014). Clinical characteristics of infections in humans due to *Staphylococcus epidermidis*. 1106 1–16. 10.1007/978-1-62703-736-5_1 24222451

[B28] Sabat A. J. Ilczyszyn W. M. van Rijen M. Akkerboom V. Sinha B. Kluytmans J. (2015). Genome-wide analysis reveals two novel mosaic regions containing an ACME with an identical DNA sequence in the MRSA ST398-t011 and MSSA ST8-t008 isolates. 70 1298–1302. 10.1093/jac/dku531 25634990

[B29] Sabat A. J. Kock R. Akkerboom V. Hendrix R. Skov R. L. Becker K. (2013). Novel organization of the arginine catabolic mobile element and staphylococcal cassette chromosome mec composite island and its horizontal transfer between distinct *Staphylococcus aureus* genotypes. 57 5774–5777. 10.1128/AAC.01321-13 24002094 PMC3811323

[B30] Shibuya Y. Hara M. Higuchi W. Takano T. Iwao Y. Yamamoto T. (2008). Emergence of the community-acquired methicillin-resistant *Staphylococcus aureus* USA300 clone in Japan. 14 439–441. 10.1007/s10156-008-0640-1 19089559

[B31] Sievert D. M. Ricks P. Edwards J. R. Schneider A. Patel J. Srinivasan A. (2013). Antimicrobial-resistant pathogens associated with healthcare-associated infections: summary of data reported to the National Healthcare Safety Network at the Centers for Disease Control and Prevention, 2009-2010. 34 1–14. 10.1086/668770 23221186

[B32] Sullivan M. J. Petty N. K. Beatson S. A. (2011). Easyfig: a genome comparison visualizer. 27 1009–1010. 10.1093/bioinformatics/btr039 21278367 PMC3065679

[B33] Talan D. A. Krishnadasan A. Gorwitz R. J. Fosheim G. E. Limbago B. Albrecht V. (2011). Comparison of *Staphylococcus aureus* from skin and soft-tissue infections in US emergency department patients, 2004 and 2008. 53 144–149. 10.1093/cid/cir308 21690621

[B34] Thurlow L. R. Joshi G. S. Clark J. R. Spontak J. S. Neely C. J. Maile R. (2013). Functional modularity of the arginine catabolic mobile element contributes to the success of USA300 methicillin-resistant *Staphylococcus aureus*. 13 100–107. 10.1016/j.chom.2012.11.012 23332159 PMC3553549

[B35] Thurlow L. R. Joshi G. S. Richardson A. R. (2012). Virulence strategies of the dominant USA300 lineage of community-associated methicillin-resistant *Staphylococcus aureus* (CA-MRSA). 65 5–22. 10.1111/j.1574-695X.2012.00937.x 22309135 PMC4090103

[B36] Urushibara N. Kawaguchiya M. Onishi M. Mise K. Aung M. S. Kobayashi N. (2016). Novel structures and temporal changes of arginine catabolic mobile elements in methicillin-resistant *Staphylococcus aureus* genotypes ST5-MRSA-II and ST764-MRSA-II in Japan. 60 3119–3122. 10.1128/AAC.02356-15 26856835 PMC4862510

[B37] Wu Z. Li F. Liu D. Xue H. Zhao X. (2015). Novel type XII staphylococcal cassette chromosome mec harboring a new cassette chromosome recombinase, CcrC2. 59 7597–7601. 10.1128/AAC.01692-15 26416872 PMC4649182

[B38] Xue H. Wu Z. Li L. Li F. Wang Y. Zhao X. (2015). Coexistence of heavy metal and antibiotic resistance within a novel composite staphylococcal cassette chromosome in a *Staphylococcus haemolyticus* isolate from bovine mastitis milk. 59 5788–5792. 10.1128/AAC.04831-14 26169408 PMC4538546

[B39] Xue H. Wu Z. Qiao D. Tong C. Zhao X. (2017). Global acquisition of genetic materials from different bacteria into the staphylococcal cassette chromosome elements of a *staphylococcus epidermidis*. 50 581–587. 10.1016/j.ijantimicag.2017.06.015 28705673

[B40] Zhang Y. Q. Ren S. X. Li H. L. Wang Y. X. Fu G. Yang J. (2003). Genome-based analysis of virulence genes in a non-biofilm-forming *Staphylococcus epidermidis* strain (ATCC 12228). 49 1577–1593. 10.1046/j.1365-2958.2003.03671.x 12950922

