# Supplementary figures and images for: Arginine Catabolic Mobile Elements in Livestock-Associated Methicillin-Resistant Staphylococcal Isolates From Bovine Mastitic Milk in China
Source: Front Microbiol. 2018 May 16;9:1031. doi: 10.3389/fmicb.2018.01031 (PMC5964201; doi:10.3389/fmicb.2018.01031)

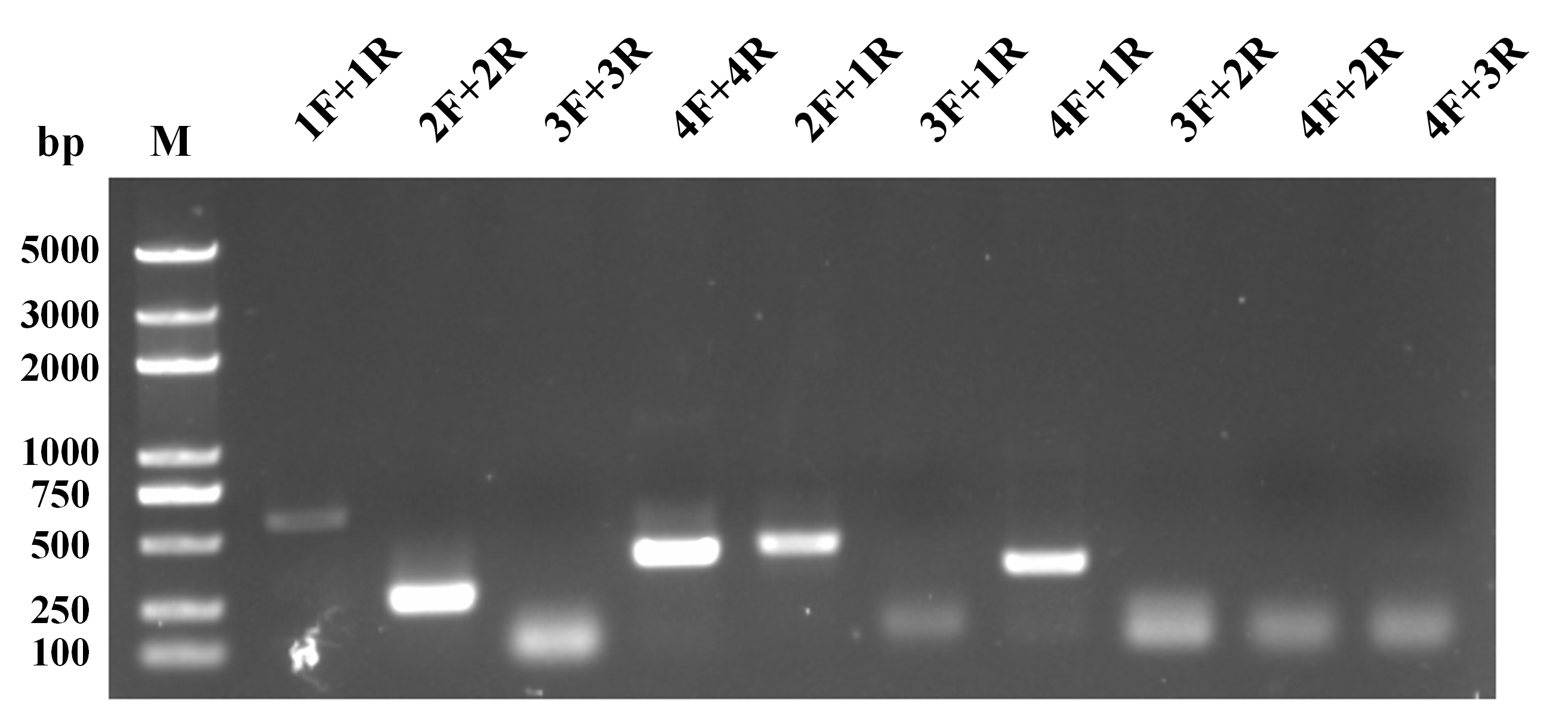

Supplement: FIGURE S1 — PCR results confirming the formation of extrachromosomal circular intermediate using different primer sets. [file Image_1.TIF]
