# Supplementary material for: Arginine Catabolic Mobile Elements in Livestock-Associated Methicillin-Resistant Staphylococcal Isolates From Bovine Mastitic Milk in China
Source: Front Microbiol. 2018 May 16;9:1031. doi: 10.3389/fmicb.2018.01031 (PMC5964201; doi:10.3389/fmicb.2018.01031)
Supplement: TABLE S1 — Oligonucleotides used in this work. [file Table_1.DOCX]

Supplementary Material

**Arginine Catabolic Mobile Elements in Livestock-Associated Methicillin-Resistant Staphylococcal Isolates from Bovine Mastitic Milk in China**

Chao Tong,^1^ Zhaowei Wu,^1^ Xin Zhao^1, 2*^, Huping Xue^1*^

*** Correspondence:** Xin Zhao: [xin.zhao@mcgill.ca](mailto:xin.zhao@mcgill.ca)

Huping Xue: [xuehuping@hotmail.com](mailto:xuehuping@hotmail.com)

**Supplementary Tables**

Table S1. Oligonucleotides used in this work.

| Primers | Sequences | Comments | Reference |
| --- | --- | --- | --- |
| 1(Forward) | AAGCACAATCCATTTCTC | closing gaps | This work |
| 1(Reverse) | TCATCATTCAACGGTCTA | closing gaps | This work |
| 2(Forward) | AAGCCAGGATAATGTAGT | closing gaps | This work |
| 2(Reverse) | ATTCCTCCAAACCATAAA | closing gaps | This work |
| 3(Forward) | AGGTGCGAAGTGTTTG | closing gaps | This work |
| 4(Reverse) | TGTCAGTCTGGCATTA | closing gaps | This work |
| 4(Forward) | AGGCTCACCTGATGTT | closing gaps | This work |
| 4(Reverse) | CAGCGAATAGTGAACC | closing gaps | This work |
| 5(Forward) | AACGAAAGCCAAGCAA | closing gaps | This work |
| 5(Reverse) | AAGTATGCTCCCAAAA | closing gaps | This work |
| 6(Forward) | ATTTGTTGCCAAGACC | closing gaps | This work |
| 6(Reverse) | AAACGGTTCCAACGAA | closing gaps | This work |
| 1R | TACCTTGTTCCCATTTAG | excision detection | This work |
| 1F | TTCTACCTTGACCTTTT | excision detection | This work |
| 2R | CTTTACATCGCTTACTG | excision detection | This work |
| 2F | AGTATGGCAATCCTTATC | excision detection | This work |
| 3R | ACATCATATTTCGCATG | excision detection | This work |
| 3F | CAATAAACACCCATTCATTT | excision detection | This work |
| 4R | GTAGAATTATGTTTAGGGAA | excision detection | This work |
| 4F | ATTGCCACGCTTTCAGT | excision detection | This work |
| *arcA*-F | CTAACACTGAACCCCAATG | *arcA* detection | (Diep et al., 2008) |
| *arcA*-R | GAGCCAGAAGTACGCGAG | *arcA* detection | (Diep et al., 2008) |
| *opp3AB*-F | GCAAATCTGTAAATGGTCTGTTC | *opp3AB* detection | (Diep et al., 2008) |
| *opp3AB*-R | GAAGATTGGCAGCACAAAGTG | *opp3AB* detection | (Diep et al., 2008) |

Table S2. Predicted ORFs in the SCC elements and ACME of *S. epidermidis* Y24

| ORFs | Location | size (bp) | Gene | Description |
| --- | --- | --- | --- | --- |
| ORF1 | 101..580 | 480 | *orfX* | LSU m3Psi1915 methyltransferase rlmh |
| ORF2 | 804..1130 | 327 |  | Transcriptional regulator, XRE family |
| ORF3 | 1167..2000 | 834 |  | CAAX amino terminal protease family |
| ORF4 | 2205..2369 | 165 |  | Hypothetical protein |
| ORF5 | 2686..3459 | 774 |  | Hypothetical protein |
| ORF6 | 4056..3697 | 360 |  | Hypothetical protein |
| ORF7 | 5291..4221 | 1071 | *fabK* | Enoyl-[acyl-carrier-protein] reductase [FMN] (EC 1.3.1.9) |
| ORF8 | 5951..5304 | 648 | *LysR* | Lysr-family regulatory protein |
| ORF9 | 6649..5975 | 675 | *IS431* | IS431 transposase |
| ORF10 | 6907..7074 | 168 | *PksG* | Hydroxymethylglutaryl-coa synthase N-domain homolog (EC 2.3.3.10) |
| ORF11 | 7951..8694 | 744 | *ugpQ* | Glycerophosphoryl diester phosphodiesterase (EC 3.1.4.46) |
| ORF12 | 8791..9219 | 429 |  | Hypothetical protein |
| ORF13 | 11271..9265 | 2007 | *MecA* | Penicillin-binding protein PBP2a, methicillin resistance determinant meca, transpeptidase |
| ORF14 | 11371..12357 | 987 | *ΔBlaR1* | Truncated Regulatory sensor-transducer, blar1/mecr1 family |
| ORF15 | 12329..12589 | 261 | *hsdR* | Type I restriction-modification system, restriction subunit R (EC 3.1.21.3) |
| ORF16 | 14100..12580 | 1521 | *IS1272* | IS1272 transposase |
| ORF17 | 14748..14239 | 510 |  | Hypothetical protein |
| ORF18 | 15071..14760 | 312 |  | Hypothetical protein |
| ORF19 | 15508..15158 | 351 |  | Hypothetical protein |
| ORF20 | 17658..16030 | 1629 | *ccrB2* | Cassette chromosome recombinase B |
| ORF21 | 19029..17680 | 1350 | *ccrA2* | Cassette chromosome recombinase A |
| ORF22 | 21050..19263 | 1788 |  | Hypothetical protein |
| ORF23 | 21340..21050 | 291 |  | Hypothetical protein |
| ORF24 | 21479..22528 | 1050 |  | Hypothetical protein |
| ORF25 | 23146..24636 | 1491 |  | Hypothetical protein |
| ORF26 | 25182..25009 | 174 |  | Zn-dependent hydroxyacylglutathione hydrolase / Polysulfide binding protein |
| ORF27 | 26264..25260 | 1005 |  | Metallo-beta-lactamase superfamily protein |
| ORF28 | 26383..26520 | 138 |  | Hypothetical protein |
| ORF29 | 26517..26888 | 372 |  | HNH endonuclease family protein |
| ORF30 | 27017..27637 | 621 |  | Hypothetical protein |
| ORF31 | 28743..28510 | 234 |  | Hypothetical protein |
| ORF32 | 28989..36872 | 7884 | *pls* | Antiadhesin Pls, binding to squamous nasal epithelial cells |
| ORF33 | 36996..38612 | 1617 | *gtfC* | Poly (glycerol-phosphate) alpha-glucosyltransferase |
| ORF34 | 40283..38772 | 1512 | *gtfD* | Poly(glycerol-phosphate) alpha-glucosyltransferase (EC 2.4.1.52) |
| ORF35 | 41964..40306 | 1659 | *murF* | UDP-N-acetylmuramoylalanyl-D-glutamyl-2,6-diaminopimelate--D-alanyl-D-alanine ligase |
| ORF36 | 42356..41958 | 399 |  | Putative, UDP-N-acetylmuramoylalanyl-D-glutamyl-2,6-diaminopimelate--D-alanyl-D-alanine ligase |
| ORF37 | 42644..42766 | 123 |  | Hypothetical protein |
| ORF38 | 43205..43834 | 630 |  | Hypothetical protein |
| ORF39 | 43849..44091 | 243 |  | Hypothetical protein |
| ORF40 | 44490..45341 | 852 |  | Mobile element protein |
| ORF41 | 46370..45441 | 930 | *arcC* | Carbamate kinase (EC 2.7.2.2) |
| ORF42 | 47388..46390 | 999 | *arcB* | Ornithine carbamoyltransferase (EC 2.1.3.3) |
| ORF43 | 48115..47426 | 690 | *arcR* | Transcriptional regulator arcr essential for anaerobic expression of the ADI pathway, Crp/Fnr family |
| ORF44 | 49578..48157 | 1422 | *arcD* | Arginine/ornithine antiporter arcd |
| ORF45 | 50899..49664 | 1236 | *arcA* | Arginine deiminase (EC 3.5.3.6) |
| ORF46 | 51614..51168 | 447 | *argR* | Arginine pathway regulatory protein argr, repressor of arg regulon |
| ORF47 | 52702..52869 | 168 |  | Hypothetical protein |
| ORF48 | 53571..53140 | 432 |  | Universal stress protein family |
| ORF49 | 54779..54453 | 327 |  | Replication protein |
| ORF50 | 54795..55469 | 675 |  | Mobile element protein |
| ORF51 | 55847..56662 | 816 |  | Hypothetical protein |
| ORF52 | 57318..56923 | 396 |  | Radical SAM domain protein |
| ORF53 | 57468..57331 | 138 |  | Hypothetical protein |
| ORF54 | 57629..58534 | 906 |  | ABC transporter ATP-binding protein |
| ORF55 | 58509..59630 | 1122 |  | Hypothetical protein |
| ORF56 | 59596..60207 | 612 |  | Hypothetical protein |
| ORF57 | 60288..60953 | 666 |  | Response regulator |
| ORF58 | 60941..62032 | 1092 |  | Two-component sensor histidine kinase |
| ORF59 | 62540..62680 | 141 |  | Hypothetical protein |
| ORF60 | 62956..64317 | 1362 |  | Sugar transporter |
| ORF61 | 64863..65348 | 486 |  | Immunodominant antigen B |
| ORF62 | 65771..67834 | 2064 | *copB* | Lead, cadmium, zinc and mercury transporting atpase (EC 3.6.3.3) (EC 3.6.3.5);  Copper-translocating P-type atpase (EC 3.6.3.4) |
| ORF63 | 67849..69282 | 1434 | *cueO* | Multicopper oxidase |
| ORF64 | 69302..69784 | 483 |  | DUF1541 domain-containing protein |
| ORF65 | 71584..70256 | 1329 |  | Zn-dependent hydroxyacylglutathione hydrolase / Polysulfide binding protein |
| ORF66 | 72673..71603 | 1071 |  | Disulfide bond regulator |
| ORF67 | 72812..73072 | 261 |  | Uncharacterized protein |
| ORF68 | 73087..73827 | 741 |  | Hypothetical protein |
| ORF69 | 74283..73888 | 396 | *arsC2* | Arsenate reductase (EC 1.20.4.1) |
| ORF70 | 75591..74302 | 1290 | *arsB* | Arsenic efflux pump protein |
| ORF71 | 75908..75594 | 315 | *arsR* | Arsenical resistance operon repressor |
| ORF72 | 77788..76058 | 1731 | *arsA* | Arsenical pump-driving ATPase (EC 3.6.3.16) |
| ORF73 | 78116..77769 | 348 | *arsD* | Arsenical resistance operon trans-acting repressor arsd |
| ORF74 | 78637..78957 | 321 | *arsR* | Arsenical resistance operon repressor |
| ORF75 | 79045..79929 | 885 |  | Transporter |
| ORF76 | 79943..80071 | 129 |  | Hypothetical protein |
| ORF77 | 80424..81209 | 786 |  | Hypothetical protein |
| SCC-DR | 562-580 | 19 | DR | Direct repeat of SCC |
| SCC-DR | 27942-27960 | 19 | DR | Direct repeat of SCC |
| SCC-DR | 43098-43116 | 19 | DR | Direct repeat of SCC |
| SCC-DR | 55661-55679 | 19 | DR | Direct repeat of SCC |
| SCC-DR | 81460-81478 | 19 | DR | Direct repeat of SCC |

Table S3 Defensive systems and origin of the strains

| Strain name | Origin | Defensive system | Accession no. |
| --- | --- | --- | --- |
| Y24 | *S. epidermidis* | Type I R-M system | KY849363 |
| M1 | *S. aureus* | Type I R-M system | HM030720 |
| M299 | *S. aureus* | Type I R-M system | HM030721 |
| ATCC 12228 | *S. epidermidis* | Type I R-M system | AE015929 |
| R99 | *S. aureus* | Type I R-M system | KF234240 |
| R15 | *S. aureus* | Type I R-M system | KF184643 |
| V2200 | *S. aureus* | Type I R-M system | CP007657 |
| JCSC1968 | *S. aureus* | Type I R-M system | AB063172 |
| 5909-02 | *S. schleiferi* | Type I R-M system | CP009676 |
| CA15 | *S. aureus* | Type I R-M system | CP007674 |
| JKD6159, | *S. aureus* | Type I R-M system | CP002114 |
| WKZ-2 | *S. aureus* | Type I R-M system | GQ918137 |
| RI46 | *S. aureus* | Type I R-M system | KM252873 |
| SA268 | *S. aureus* | Type I R-M system | CP006630 |
| USA300-ISMMS1 | *S. aureus* | Type I R-M system | CP007176 |
| 5118.N | *S. aureus* | Type I R-M system | CP016855 |
| USA300_2014.C01 | *S. aureus* | Type I R-M system | CP012119 |
| MW2 | *S. aureus* | Type I R-M system | BA000033 |
| SR 388 | *S. aureus* | Type I R-M system | AB665981 |
| SA1213 | *S. aureus* | Type I R-M system | AB721404 |
| 5sau489 | *S. aureus* | Type I R-M system | KX232516 |
| MSHR1132 | *S. argenteus* | Type I R-M system | FR821777 |
| DY39 | *S. warneri* | Type I R-M system | KU170612 |
| UTSW MRSA 55 | *S. aureus* | Type I R-M system | CP013231 |
| 25b_MRSA | *S. aureus* | Type I R-M system | CP010299 |
| 26b_MRSA | *S. aureus* | Type I R-M system | CP010298 |
| 33b | *S. aureus* | Type I R-M system | CP010297 |
| 31b_MRSA | *S. aureus* | Type I R-M system | CP010296 |
| 29b_MRSA | *S. aureus* | Type I R-M system | CP010295 |
| UA-S391_USA300 | *S. aureus* | Type I R-M system | CP007690 |
